# An ellipsometric approach towards the description of inhomogeneous polymer-based Langmuir layers

**DOI:** 10.3762/bjnano.7.107

**Published:** 2016-08-08

**Authors:** Falko O Rottke, Burkhard Schulz, Klaus Richau, Karl Kratz, Andreas Lendlein

**Affiliations:** 1 Institute of Biomaterial Science and Berlin-Brandenburg Centre for Regenerative Therapies (BCRT), Helmholtz-Zentrum Geesthacht, Kantstraße 55, 14513 Teltow, Germany; 2 Institute of Chemistry, University of Potsdam, Karl-Liebknecht-Straße 24–25, 14476 Golm, Germany

**Keywords:** ellipsometric mapping, Langmuir monolayer, polyester, root mean square roughness, spectroscopic ellipsometry

## Abstract

The applicability of nulling-based ellipsometric mapping as a complementary method next to Brewster angle microscopy (BAM) and imaging ellipsometry (IE) is presented for the characterization of ultrathin films at the air–water interface. First, the methodology is demonstrated for a vertically nonmoving Langmuir layer of star-shaped, 4-arm poly(ω-pentadecalactone) (PPDL-D4). Using nulling-based ellipsometric mapping, PPDL-D4-based inhomogeneously structured morphologies with a vertical dimension in the lower nm range could be mapped. In addition to the identification of these structures, the differentiation between a monolayer and bare water was possible. Second, the potential and limitations of this method were verified by applying it to more versatile Langmuir layers of telechelic poly[(*rac*-lactide)-*co*-glycolide]-diol (PLGA). All ellipsometric maps were converted into thickness maps by introduction of the refractive index that was derived from independent ellipsometric experiments, and the result was additionally evaluated in terms of the root mean square roughness, *R*_q_. Thereby, a three-dimensional view into the layers was enabled and morphological inhomogeneity could be quantified.

## Introduction

Ellipsometry is an established in situ technique to investigate surfaces, capable to derive information about dielectric properties of a film and its thickness [1]. Like applications of thin films, the uses of ellipsometry are widespread, ranging from the characterization of metal-oxide layers in semiconductors, over the characterization of functional organic films, to investigations of biologically relevant processes such as bioadsorption [2–4].

Ellipsometry is based on the phenomenon that light undergoes a change in its polarization state after reflection at a surface [5]. In an ideal situation of an isotropic, perfectly planar surface, this change in polarization can be evaluated by ellipsometric angles Δ and Ψ, related to the Fresnel reflection coefficients *r*_p_ and *r*_s_ for light of p and s polarization, respectively, which are related by tan(Ψ)exp(iΔ) = *r*_p_/*r*_s_. However, for a realistic application, the ambient and the substrate must be taken into account. Additionally, samples can be rough, inhomogeneous or anisotropic. Considering this, the overall complex reflection coefficients are measured as a function of the angle of incidence, φ, and wavelength, λ, as


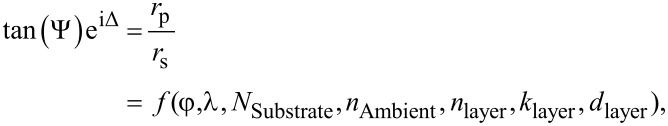


where *N*_Substrate_ are the optical constants of the substrate, *n*_Ambient_ is the refractive index of the ambient medium, and *n*_layer_, *k*_layer_ and *d*_layer_ represent the sample’s refractive index, absorption properties and layer thickness, respectively.

With subsequent modeling of primary data, material properties such as film thickness, *d*, refractive index, *n*, or even the dielectric function, ε, may be obtained. However, for ultrathin films there is almost no change in Ψ, which decreases the number of independent variables down to Δ only and makes the simultaneous and unambiguous determination of *d* and *n* impossible [6].

Various, significant accomplishments have led to the development of special types of ellipsometry. Starting from Drude’s pioneering work, the basic principles of ellipsometry have not changed, but its measurement modes have expanded. The main developments of ellipsometry are represented by spectroscopic ellipsometry (SE) and various angles spectroscopic ellipsometry (VASE), all of them developed to improve the performance of ellipsometry and to expand the application of ellipsometry towards non-ideal cases like inhomogeneous and/or rough samples. With the increasing industrial demand for chemically and/or topographically inhomogeneous films and coatings, imaging techniques have become highly appreciated. For the air–water interface, Brewster angle microscopy (BAM) is a well-established technique to visualize the topography of a layer [7], but it is also possible to use imaging ellipsometry (IE) [8]. Both are classical imaging techniques with respect to the principle of simply visualizing reflection intensities. Indeed, this approach provides fine images of thin films, laterally structured at the µm scale. But, a quantitative evaluation by BAM is difficult, as it requires an intense calibration procedure [9]. Besides these common imaging techniques, it is also possible to use ellipsometric mapping, a method that is not based on photometric principles like BAM and IE are [10]. The basic idea of ellipsometric mapping is to visualize the distribution of the ellipsometric angles Δ and Ψ over a certain region. Moreover, it is even possible to generate thickness maps with lateral dimension at the µm scale and vertical dimension at the nm scale. Additionally, the root mean square roughness, *R*_q_, of an observed surface can be evaluated. This, in combination with the dispersion of Δ and Ψ over a region of interest (ROI), allows easy differentiation between various parts of an inhomogeneous layer [11]. In any case, both techniques, IE and mapping, require a spatially resolving detector, most commonly a CCD camera. In recent years, the use of ellipsometric mapping techniques has continued to increase, helping researchers to investigate structured silicon wafers, spin-coated films or even antibody–antigen interactions on structured substrates [12–14].

However, up to now, all reports concerning ellipsometric mapping are limited to applications on solid substrates, except for one work, in which a fatty acid based, collapsed Langmuir layer was investigated [15]. The inhomogeneity of the collapsed layer could be displayed by mapping the ellipsometric angle Δ, and was evaluated by statistical analysis of all micrograph pixels. The infrequent application of ellipsometric mapping at the air–water interface might be caused by the peculiarities of the air–water interface: In contrast to thin films on solid substrates, one will observe lateral movement for almost every surfactant film. This movement originates from thermal and/or surface pressure gradients or even from air draft. This movement does not significantly hinder the recording of a fully focused IE or BAM image, which takes about one second; whereas it takes several tens of seconds (depending on chosen measurement parameters) to collect an ellipsometric map. The negative effects of lateral film movement on the recording of ellipsometric maps can be seen in [16], as the generated Δ-maps were riddled by artefacts in the form of structural elements that are displayed two or more times within one ellipsometric map. Moreover, layers at the air–water interface are well within the ultrathin film limit and, as it has been ruled out earlier, this hampers modeling. Lastly, the use of solid substrates increases the optical contrast in many cases. Nevertheless, in situ studies of certain compounds at the air–water interface are often desired.

For the present report, we hypothesize that ellipsometric mapping can be applied successfully for ultrathin polymeric layers at the air–water interface and provides an elegant way to investigate and quantify morphological inhomogeneities. The prerequisites, required adjustments and limitations are presented with suitable examples.

As a proper compound for ellipsometric mapping studies, a system was required with low or no lateral movement at the air–water interface, being close to a solid substrate and hence, simplifying first mapping attempts. Moreover, a distinct morphological inhomogeneity would be desirable to use the power of mapping to full capacity. Considering these requirements, a poly(ω-pentadecalactone) (PPDL)-based Langmuir layer was chosen, as it fulfills the demands of the absence of lateral movement and pronounced structuration. Additionally, the demonstration of spectroscopic ellipsometry on PPDL-based Langmuir layers has already been shown [17].

Morphological transitions that lead to inhomogeneous structures can be caused by a plethora of compounds at the air–water interface and are known for fatty acid based layers [18], as well as for those consisting of phospholipids [11], polymers [19], and even proteins [20]. All of them are amphiphilic in character and nonabsorbing in the visible spectral range, similar to PPDL, making the latter an appropriate candidate to represent this group of compounds.

## Results and Discussion

**Figure 1:**
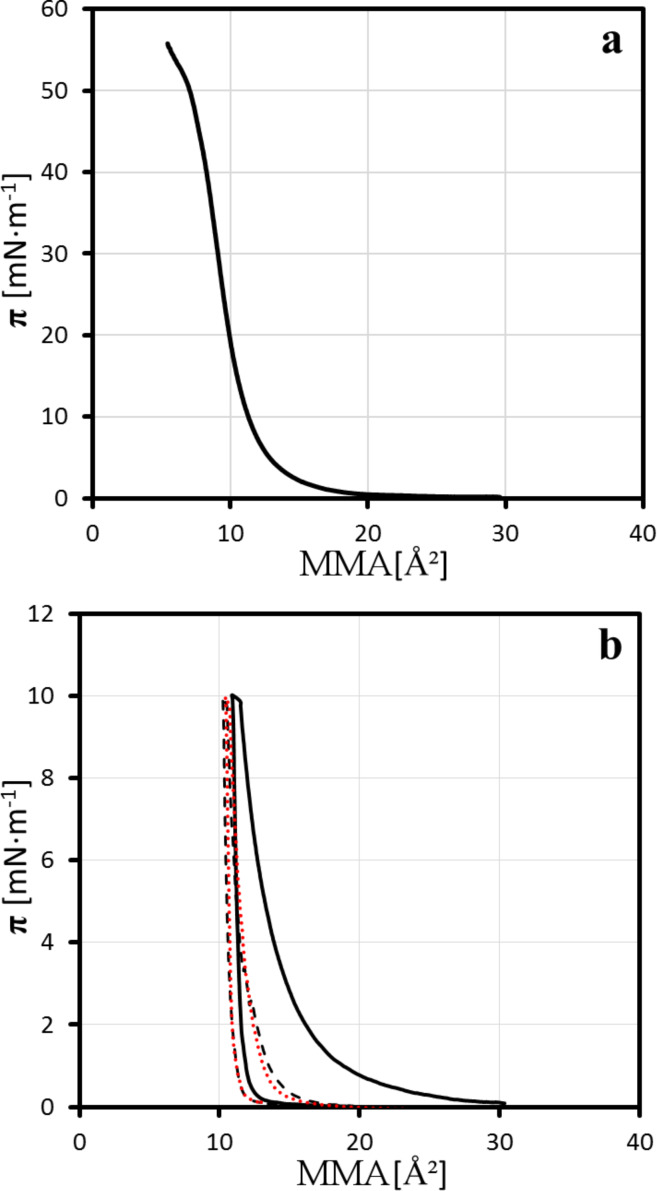
π–*A* isotherm (a) and compression–expansion curve (b) of PPDL-D4 recorded on water at room temperature. MMA is the mean molecular area per monomer unit. For the compression–expansion curve, the black, dashed-black and red-dotted lines are the first, second and third compression–expansion cycles, respectively.

For presentation and discussion of mapping results, the Langmuir characterization of the PPDL-D4-based layers is necessary because this specific compound has not been previously investigated by the Langmuir technique. Its surface pressure–area (π–*A*) isotherm and compression–expansion isotherms are shown in Figure 1. The π–*A* isotherm is identical to that of an already investigated PPDL-based Langmuir layer, namely OPDL–TMDI [17], which consists of oligo(ω-pentadecalactone) segments linked by the urethane linker TMDI (2,2,4-trimethyl-hexamethylene diisocyanate).

**Figure 2:**
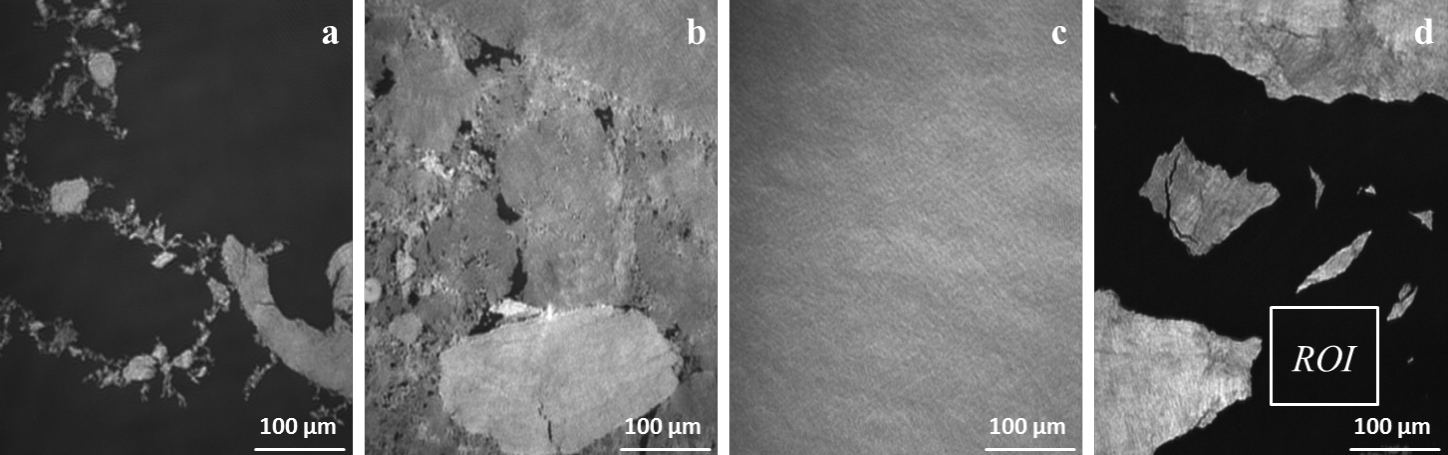
Brewster angle microscopy images of star-shaped PPDL-D4 at different compression states recorded on water at 22 °C. (a) 0.0 mN·m^−1^; (b) 0.8 mN·m^−1^; (c) 10 mN·m^−1^; (d) film rupture after stress release (imaging ellipsometry).

The mean molecular area (MMA) values found for the initial increase in the compression isotherm are clearly smaller as expected. The reason is the unusual spreading behavior of the polymer: Directly after spreading, without any increase in the surface pressure, irreversibly formed floe-like structures can be observed by BAM and IE (Figure 2a). The short time elapsed between the spreading procedure and the observation of morphologies leads to the assumption that they are generated by precipitation-like processes. During compression, the free areas in between the morphologies are reduced, causing just a small increase of surface pressure. When only a few, small free spots are left, the surface pressure begins to increase quiet rapidly (Figure 2b). Further compression leads to a closed film (Figure 2c).

The lack of film movement during compression of the PPDL-D4 Langmuir layers should also be noted. This behavior can be observed at the state of a closed layer at high pressure (Figure 2c), but also for the inhomogeneous films (Figure 2a,b). By stopping the barriers' movement, the film’s lateral movement is impeded additionally. For further investigation compression–expansion experiments were performed. In this way a distinct shift was observed for PPDL-D4 between the first and second compression–expansion cycle, indicating that the compression–expansion cycle is accompanied by a loss of material into the substrate or an irreversible reorganization process. Regarding Figure 2d, which reflects the morphology after the first expansion, it becomes clear that the stress release results in film rupture. Surprisingly, those structures then move laterally on the water surface, leading to the question of what caused the lack of movement after spreading and during compression and what has changed during the expansion part of the experiment.

**Figure 3:**
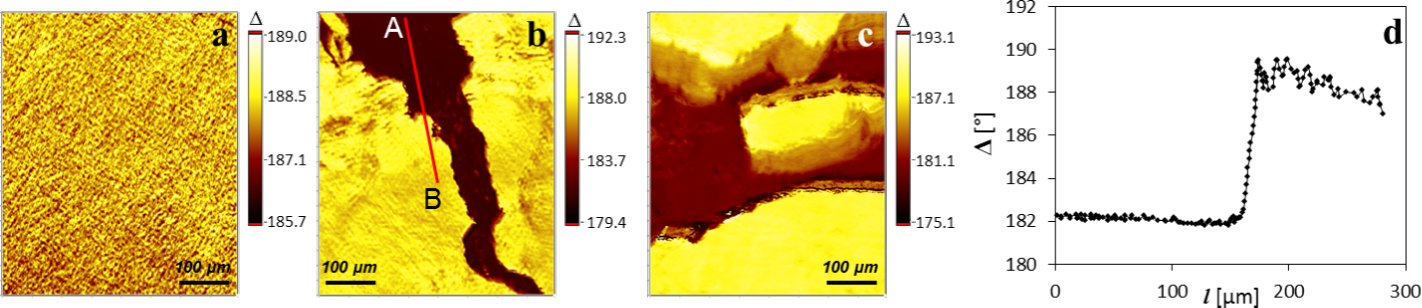
(a–c) Maps of the ellipsometric angle, Δ, recorded on water at room temperature. (a) Represents Δ-values for a closely packed PPDL-D4 film at 10 mN·m^−1^. (b) Recorded before the film was closed by compression and (c) after the film rupture due to stress release. (d) Represents the cross-section trace that can be seen in (b) as red line (A and B indicate the start and end of the cross-section).

However, the lack of lateral movement during compression lends PPDL-D4 Langmuir layers for observation by nulling-based ellipsometric mapping, as one ellipsometric mapping run takes several tens of seconds, depending on the chosen parameters. In contrast to BAM and IE, which provide 2D images of laterally resolved reflection intensities at one specific polarizer, compensator and analyzer setting, the idea of nulling-based ellipsometric mapping is to visualize spatially resolved values of the ellipsometric angles Δ and/or Ψ. To obtain maps of the ellipsometric phase difference Δ, the ellipsometric null as a net value of the current ROI has to be first estimated. Subsequently, a series of ellipsometric images are taken whereby the polarizer rotates in defined steps next to the null. This procedure allows subsequent determination of the ellipsometric null for every ROI pixel. To achieve high accuracy for the determination of Δ-values, it is advisable to collect a large number of images that are recorded within the appropriately chosen polarizer range. By introducing the refractive index *n* obtained from an independent (e.g., VASE) measurement, it is possible to convert Δ into thickness maps and therefore to achieve quantitative analysis of the 3D morphology of the Langmuir layers with lateral resolution at the µm scale. Exploiting the nulling-based ellipsometric contrast in this way avoids the methodological problems inherent in the photometric evaluation of BAM or IE images and also leads to information concerning layer thickness [9]. As an example, for PPDL-D4 with 25 images and 60 frames along a polarizer range of 5° it takes approximately 20 s to record one Δ-map. The polarizer setting of 5° is based on our expectation of small differences in the height, e.g., thickness at the nm scale. In the present work only Δ-maps were recorded because of their high sensitivity towards thickness variations, even at ultrathin film conditions [6]. The complications and theoretical problems of estimating extremely small dispersions of Ψ for ultrathin films is known and well-described [5,21]. Using ellipsometric mapping, it is now possible to simultaneously investigate the floe-like structures and their surroundings. To accomplish this, a map of the ellipsometric phase shift Δ was recorded for an area comparable in size to BAM or IE images (400 × 500 µm^2^). Examples of such maps are summarized in Figure 3, proving the applicability of ellipsometric mapping to characterize ultrathin films at the air–water interface.

Without further data treatment, the first interesting, yet surprising, information can be provided by taking a detailed look at the raw data. In Figure 3a, a PPDL-D4 closed layer of slabs at a surface pressure of 10 mN·m^−1^ is shown. Within the observed area, Δ varies between 186–190°, indicating a rough, inhomogeneous surface. This is reflected in the root mean square roughness, *R*_q_, for the different regions, which is calculated from the profile of the cross-section A–B as the root mean square average of the roughness profile ordinates. Besides the analysis of the slabs it is also worthwhile to take a look at the surroundings, which are also mapped. Figure 3b shows a PPDL-D4 layer at a point during compression where there are still some “free areas” left. The Δ-values traced along the red line starting from A to B are outlined in Figure 3d. But the more surprising details are revealed by the Δ-values found in the surroundings: These are in the range of 182.0 ± 0.1°, evidencing that the slabs are not surrounded by water, but by a layer, most likely a monolayer. Applying the same measurement after the compression–expansion cycle (resulting in film rupture) also yielded interesting information. However, from a quick glance at Figure 3c it is obvious that the enhanced movement of the structures hampers the measurement caused by its static time requirement of approximately 20 s. Evaluation of the new surroundings results in a value close to 180°, suggesting that there is only water present instead of a layer on water. However, the blurry map left some uncertainty and was therefore additionally checked by the standard determination of Δ as a net value over a certain ROI. To get a clear understanding of the surroundings, the ROI was adjusted accordingly (Figure 2d). This reduces the time requirement in a way that Δ can be determined undisturbed, resulting in a value of 180.00 ± 0.05°, fitting perfectly with the calibration for water, which was executed before every measurement run and moreover confirms the results provided by the blurry Δ-map (Figure 3c). Combining now the latest results with those provided by the compression–expansion experiments, it seems likely to interpret the strong shift between the first and second compression–expansion cycle as a material loss. If we assume that the monolayer consists of PPDL-D4, it is imaginable that during compression, the monolayer collapses and/or attaches to the slabs due to attractive interaction. Indeed the monolayer composition is unknown, but an attractive interaction between the monolayer and slab supports the assumption that both consist of PPDL strains.

To exploit the possibilities of nulling-based ellipsometric mapping, and to elucidate the nature of a PPDL-D4-based Langmuir layer, the refractive index was introduced to convert Δ into thickness maps. The refractive index used for this conversion was also derived by VASE for two different surface pressures at the air–water interface. Therefore, the ROI was selected on areas without differing surroundings but only on the slabs. A simple model consisting of water, a polymeric layer and air was applied to fit the obtained data. PPDL-D4 layers were considered to be nonabsorbing over the tested spectral range (408–902 nm). For all cases, the thickness of the slabs was determined to be almost surface-pressure-independent (Table 1).

**Table 1:** Film thickness, *d*, and Cauchy coefficients of the refractive index, *n*(λ), derived from VASE measurements of PPDL-D4 Langmuir layers where RMSE is the root mean square error.

π [mN·m^−1^]	*N*_0_	*N*_1_ [nm²]	*d* [nm]	RMSE

0.5	1.524 ± 0.017	5467 ± 157	4.2 ± 0.5	0.140
10	1.531 ± 0.019	0	4.3 ± 0.5	0.144

**Figure 4:**
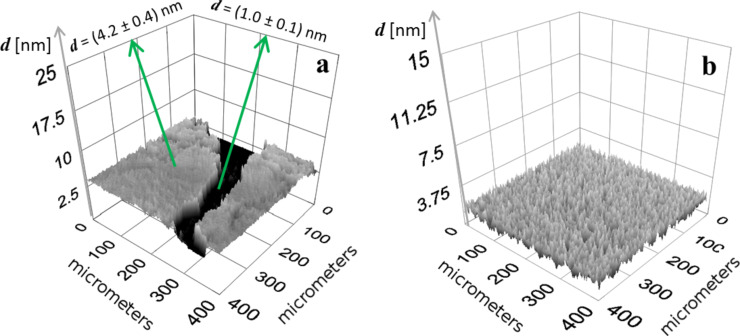
Thickness maps of PPDL-D4 (a) and PLGA (b) derived from Δ-maps corresponding to Figure 3b and Figure 6b, respectively. For PPDL-D4, the thickness values for the slabs of around 4 nm and 1 nm for the surrounding layer were obtained.

For the generation of thickness maps, which reveal the vertical dimension of the slabs and the monolayer, the refractive index determined at 10 mN·m^−1^ was applied. A thickness map resulting from the described procedure is shown in Figure 4. The slab thickness was found to be 4.2 ± 0.4 nm, whereas the surrounding layer thickness was around 1.0 ± 0.1 nm and is comparatively very smooth. This result confirms our assumption drawn from Δ-maps that the surrounding layer should be considered to be a monolayer. Moreover, the rough surface of the slabs can be elucidated upon first glance. However, for both Δ-maps and thickness maps, the roughness can be evaluated in the form of root mean square roughness, *R*_q_, which is discussed later. However, in any case, the values must be handled with some care, keeping in mind that at ultrathin film conditions, the film thickness and refractive index are strongly coupled. For a qualitative assessment, the introduction of even approximate refractive indices can be useful [22].

**Figure 5:**
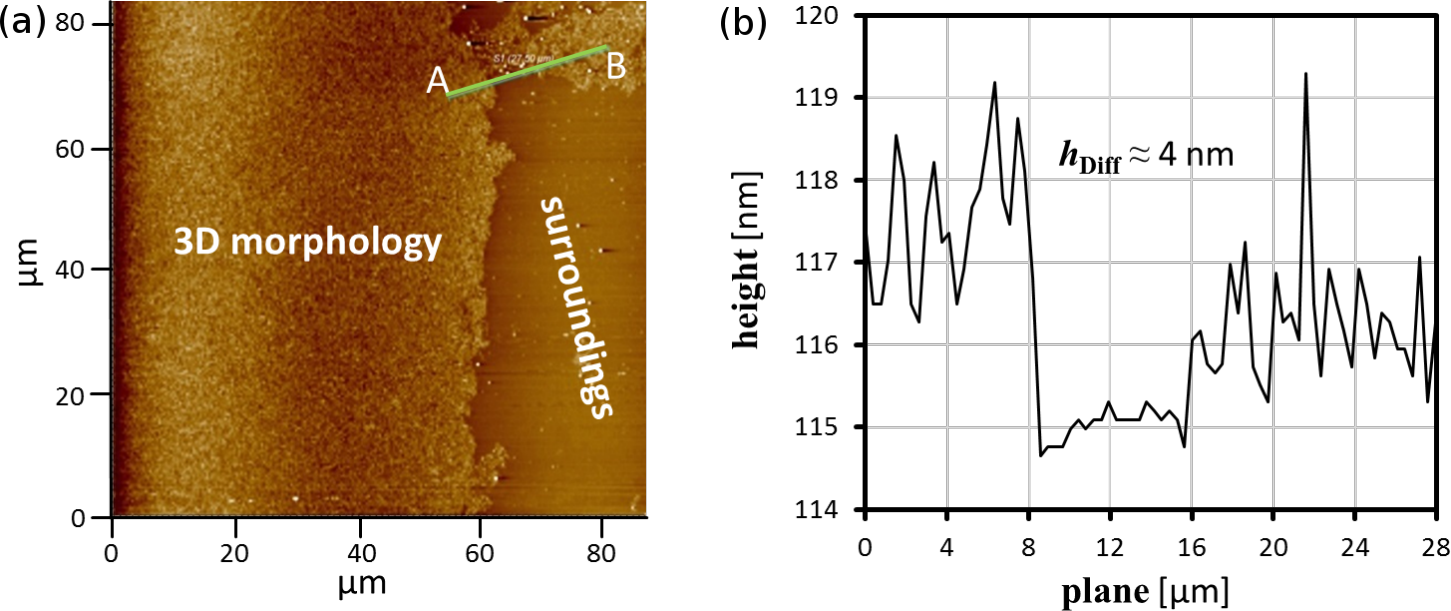
(a) AFM images of a PPDL-D4 LB layer transferred onto mica at 0.5 mN·m^−1^. (b) Height diagram of the cross-section trace along the green line starting from A, ending at B where *h*_Diff_ is the height difference.

To prove the presented results, AFM investigations of transferred Langmuir-Blodgett (LB) films were also performed. The Langmuir layers of PPDL-D4 were transferred onto mica at a surface pressure of 0.5 mN·m^−1^. The surface pressure was chosen at such a low value to be sure that a mixture of slabs and monolayer was still present. The dewetted LB films were subsequently scanned for regions where the slab’s edge was visible, so that the height difference from slab to monolayer could be measured. Several spots of such topography were found, as shown for example in Figure 5.

Regarding Figure 5b, the height gap between the slab and monolayer is around 4 nm in the dewetted state. This clearly supports the results provided by the complementary use of spectroscopic ellipsometry and nulling-based thickness mapping.

**Figure 6:**
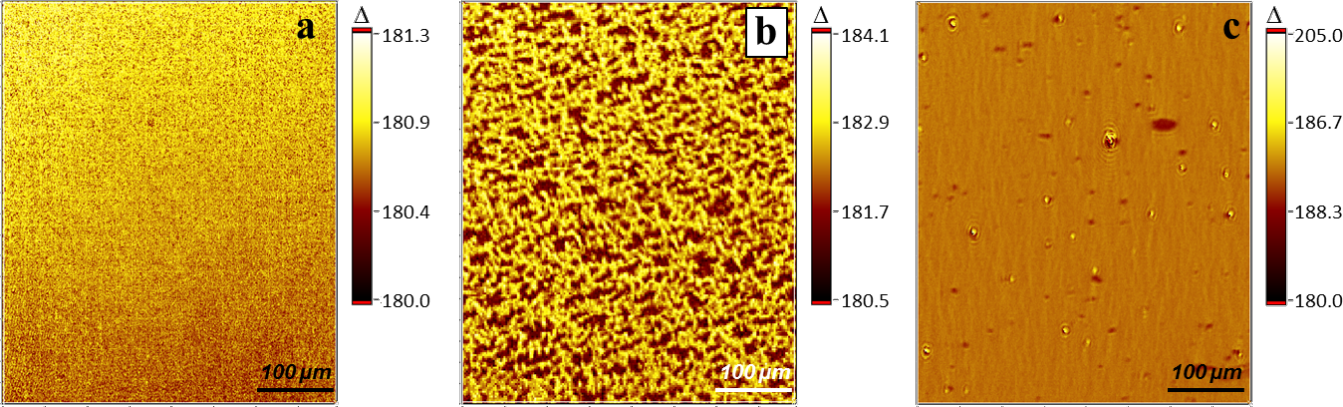
Surface-pressure-dependent structuration of PLGA-based Langmuir layers observed by ellipsometric Δ mapping at room temperature at (a)10 mN·m^−1^, (b) 12 mN·m^−1^ and (c) 25 mN·m^−1^, respectively.

Hence the low lateral movement and precipitation-caused structures of PPDL-based Langmuir layers are quite a unique system and to elucidate the limits for the applicability of this methodology, ellipsometric mapping was also applied to a telechelic poly[(*rac*-lactide)-*co*-glycolide]-diol (PLGA) layer. PLGA reversibly forms network-like structures above a certain surface pressure of 11 mN·m^−1^ [19,23]. At the state of network generation there was still significant film movement and only blurry maps were obtained. However, after some adjustments, it was indeed possible to record sharp Δ-maps. At first, the measurement time was reduced by variation of specific parameters (e.g, the number of images taken was reduced stepwise). It was found that the use of only 9 images still gave satisfying maps, which was of course accompanied by a reduction in quality. Additionally, the number of frames was also reduced; in this case, halved from 60 to 30. Altogether these adjustments lowered the measurement time to less than 10 sec. However, the main effort was achieved by suppressing almost any lateral film movement. This could be achieved by deactivation of barrier movement or in the best case by controlling the investigated system in such a way that the targeted surface pressure coincidences with the minimal area of the Langmuir trough. In this way maps of the ellipsometric phase shift Δ could be successfully recorded also for more versatile morphologies. This establishes ellipsometric mapping as an additional technique, next to BAM, for the in situ investigation of PLGA-based, surface-pressure-induced network formation (Figure 6b). In [19] it was shown that by further compression up to a surface pressure above 15 mN·m^−1^ the PLGA Langmuir layer becomes optically homogeneous. This effect could also be observed by ellipsometric mapping (Figure 6c) and could additionally be evaluated using the root mean square roughness, *R*_q_. Therefore, the obtained Δ-maps of PLGA-based Langmuir layers were converted into thickness maps by introducing a refractive index of 1.48 (Figure 4b), which was taken from [19].

**Figure 7:**
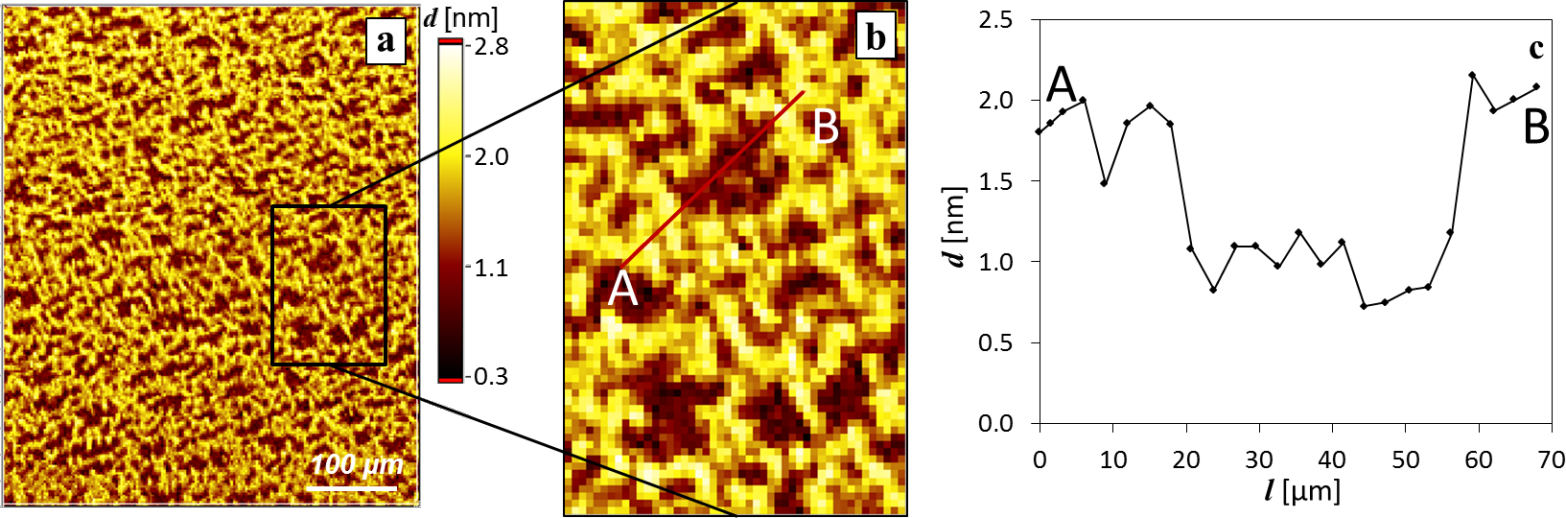
(a) Thickness map of PLGA network structures at 12 mN·m^−1^ converted from the corresponding Δ-map in Figure 6b. (b) The section that is used for a (c) cross-section trace (red line).

*R*_q_ is determined along a cross-section trace by evaluation of pixels along this path. For comparison, PPDL-D4 layers were also evaluated in this way and surface-pressure-dependent differences in topography of PLGA-based layers could be estimated well. At 10 mN·m^−1^ a roughness of 0.1 nm is determined, increasing to 0.4 nm at 12 mN·m^−1^ (network-like structure, Figure 4b and Figure 6b) and again decreasing with further compression to 0.2 nm at 25 mN·m^−1^ (Figure 6c). In the case of PPDL-D4, the roughness of the slabs was determined with 0.3 nm and was found to be surface-pressure-independent in the investigated range of 0 to 10 mN·m^−1^. For the monolayer between the slabs, an *R*_q_ of 0 to 0.1 nm was observed, fitting well to the roughness determined for the homogeneous PLGA monolayer. In addition to evaluating the roughness, the conversion into thickness maps enables investigation of even the gaps in the network-like structure. This conversion is presented in Figure 7.

From the cross-section trace evaluated over one comb of the network, it becomes obvious that the gaps are not empty and that there is still material left. As a result, we deduce that the network structures of PLGA arise from the monolayer without consuming it completely at this point of compression. This proves the applicability of nulling-based thickness mapping for more versatile Langmuir layers and underlines the value of the presented ellipsometric approach.

## Conclusion

Under certain considerations, nulling-based ellipsometric mapping can be used to characterize morphological inhomogeneities of polymer-based Langmuir layers, despite the peculiarities of the air–water interface. This is exemplified for polyester-based layers. Generally, the lower the lateral mobility of the investigated Langmuir film, the more accurate maps of the ellipsometric phase shift Δ can be obtained as a basis of more sophisticated evaluations.

For a laterally nonmoving Langmuir layer of PPDL-D4, which is characterized by irregular floe-like structures by BAM, it is possible to extract the film thickness, *d*, and its variation over larger areas at the µm scale in terms of root mean square roughness, *R*_q_, with complementary use of VASE measurements. Concerning the surroundings of the pronounced PPDL-D4-based morphologies, it is possible to differentiate between a monolayer and the bare substrate water.

By proper adjustment of the experimental parameters, it is possible to extend the applicability of this methodology to mobile Langmuir layers of PLGA, which are characterized by pressure-driven network evolution. For that purpose, the number of images and frames must be reduced and the layer mobility must be restrained to apply ellipsometric Δ mapping successfully for more mobile films.

In any case, it is advantageous to use complementary IE or BAM, still being the fastest methods to derive qualitative images of the surface. Nevertheless, ellipsometric mapping represents a straightforward way to obtain quantitative information of the layer's vertical dimension and its lateral variation over several hundred micrometers. The conversion into thickness maps allows the additional quantification of observed morphologies of certain regions within one map. This helps to investigate and describe inhomogeneous Langmuir layers. Such applications are definitely not limited to polymer-based Langmuir films. It is our expectation that achievement of a comparable or even a greater impact by ellipsometric mapping is possible for analysis of layers consisting of proteins, enzymes or other biomolecules. Furthermore, the vertical dimension of their monolayers may even exceed the ultrathin film limit, which would allow reasonable Ψ mapping.

## Experimental

### Materials

**Figure 8:**
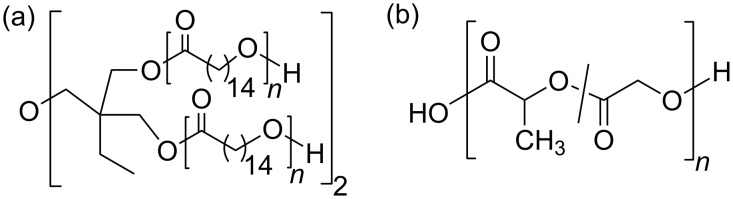
Structural description of (a) PPDL-D4 and(b) PLGA.

Both investigated polymeric compounds, star-shaped, 4-arm poly(ω-pentadecalactone) (PPDL-D4) and telechelic poly[(*rac*-lactide)-*co*-glycolide]-diol (PLGA), were synthesized according to literature methods [19,24–25] and are shown in Figure 8.

In the following a short summary of the methods and the characterization of the polymers are given. PPDL-D4 was synthesized by tin(II)-mediated ring-opening polymerization of ω-pentadecalactone and di(trimethylolpropane) as the core segment.

For PPDL-D4 a molecular weight *M*_n_ of 5000 g·mol^−1^ was determined by end group titration. PLGA was synthesized by ring-opening copolymerization of *rac*-dilactide and diglycolide using 1,8-octanediol as an initiator and dibutyltin(IV) oxide as a catalyst. The resulting PLGA had a mean molecular weight *M*_n_ of 8300 g·mol^−1^, which was evaluated by GPC (universal calibration).

For all compounds chloroform (Carl Roth, HPLC grade) was used as a spreading solvent. Ultrapure water with a nominal resistivity of ρ = 18.2 MΩ cm (Milli-Q gradient A-Merck Millipore) was used for all experiments and even for the final cleaning of the Langmuir trough and barriers.

### Langmuir trough and polymeric layers

For fabrication of Langmuir layers, a rectangular Langmuir trough (KN1006, KSV NIMA, Espoo, Finland) with a total area of 841 cm^2^ was used. The surface pressure, π, was measured via the Wilhelmy technique, whereby the Wilhelmy paper plate was located equidistant between the barriers. The whole setup, including the ellipsometer, was placed on an active vibration isolation system (halcyonics variobasic 40, Accurion, Göttingen, Germany) and covered with a laser safety cabinet, which also prevented dust from entering the experiment. Before each experiment, the Langmuir trough and its barriers were properly cleaned with chloroform and afterwards rinsed several times with water. Prior to spreading, the cleanliness of the pure water surface was checked by BAM and it was ensured that the surface pressure did not exceed 0.1 mN·m^−1^ during compression. For the formation of Langmuir layers, the sample solution was added dropwise onto the clean water surface using a 250 µL microsyringe (825 RN SYR, Hamilton Co., Reno, USA). The concentration of the spreading solutions varied from 0.25–0.35 mg·mL^−1^. The spreading procedure was followed by 10 mins for evaporation of chloroform. All Langmuir layers were compressed or expanded at a constant rate of 10 mm·min^−1^. The surface pressure was recorded as a function of the mean molecular area per repeating unit, MMA. For PPDL-D4, 240.0 g·mol^−1^ was used as the mean molecular weight. For PLGA, 67.1 g·mol^−1^ was calculated as sum of the molecular weights corresponding to lactide and glycolide units, each multiplied with the respective percentage.

Hysteresis (compression–expansion) experiments for PPDL-D4 were executed by the following procedure: compression to target pressure (10 mN·m^−1^), pressure held constant for 10 min and afterwards the barriers are reopened to the starting position.

### Spectroscopic ellipsometry

We performed variable angle spectroscopic ellipsometry (VASE) using a spectroscopic imaging ellipsometer (nanofilm_ep3, Accurion, Göttingen, Germany) with a Xe arc lamp as a light source. Within one VASE measurement, the angle of incidence (AOI) varied from 50–52° in 0.5° steps and ten discrete wavelengths (bandwidth ±8 nm) between 408–902 nm were used. To gain absolute values of Δ and ψ, all four zones were analyzed. These measurements were executed at two different surface pressures varying between 0.5 and 10 mN·m^−1^. The evaluation of the results was performed with nanofilm_ep4 evaluation software (Accurion, Göttingen, Germany). For extracting the layer thickness, *d*, and especially the refractive index, *n*, of the polymeric layer, a simple model of water–layer–air was applied, assuming the layer to be homogeneous and isotropic. Hence, since the optical properties of air and water (*n* = 1.333) are known, the required parameters of the Langmuir layer can be derived from VASE measurements. The dispersion of the Langmuir layers refractive index, *n*(λ), was described as *n*(λ) = *N*_0_ + *N*_1_/λ^2^ + *N_2_*/λ^4^, where λ is the wavelength and *N*_i_ are the Cauchy coefficients with *N*_2_ = 0.

#### Brewster angle microscopy and imaging ellipsometry

Both BAM and ellipsometric images on a ROI with a maximum area of 500 × 400 µm^2^ were obtained by the nanofilm_ep3 ellipsometer. A 658 nm class IIIB laser source in combination with a ×10 magnification lens and a CCD camera (768 × 572 pixel) were used to take all micrographs, with a resulting lateral resolution of ≈2 μm.

#### Nulling-based thickness mapping

The same ellipsometer as mentioned above was used for ellipsometric mapping. An AOI of 50° and a 658 nm laser as a light source were used. For the development of an ellipsometric map, the ellipsometric null as a net value of a current ROI was first estimated. Afterwards the polarizer was adjusted to determine the null position (5° if not noted otherwise). The number of images taken along the polarizer rotation range was 25, and the number of frames taken was 60 for the PPDL-based layers. For PLGA, those values varied and are presented and discussed in detail in the Results and Discussion section. The subsequent conversion to thickness maps was carried out with the corresponding nanofilm_ep4 software. The root mean square roughness, *R*_q_, was determined along a cross-section trace on a thickness map, whereby all pixels along the trace are taken into account for the determination.

#### Preparation of Langmuir–Blodgett (LB) films and atomic force microscopy (AFM)

LB films were prepared according to the procedure described elsewhere [17] by using mica as a substrate at low surface pressure (0.5 mN·m^−1^) and a transfer speed of 2 mm·min^−1^. A Solver PRO-M (NT-MDT, Moscow, Russia) was used to obtain the AFM images of the topography as well as phase contrast images and height profiles of the LB film surface. All measurements of LB layer samples were performed in semicontact mode under ambient conditions. Silicon cantilevers NSG10-NT-14DT with a tip radius of approximately 5 nm were used.
